# Inter-centre comparison of data on surgery and speech outcomes at 5 years of age based on the Swedish quality registry for patients born with cleft palate with or without cleft lip

**DOI:** 10.1186/s12887-022-03367-2

**Published:** 2022-05-23

**Authors:** Kristina Klintö, Marie Eriksson, Avni Abdiu, Karin Brunnegård, Jenny Cajander, Emilie Hagberg, Malin Hakelius, Christina Havstam, Hans Mark, Åsa Okhiria, Petra Peterson, Kristina Svensson, Magnus Becker

**Affiliations:** 1 Department of Clinical Sciences in Malmö, Lund University, Malmö, Sweden; 2 Department of Statistics, USBE, Umeå University, Umeå, Sweden; 3 Department of Hand Surgery, Plastic Surgery and Burns and Department of Clinical and Experimental Medicine, Linköping University, Linköping, Sweden; 4 Department of Clinical Sciences, Speech and Language Pathology, Umeå University, Umeå, Sweden; 5 Department for Surgical and Perioperative Sciences, Umeå University, Umeå, Sweden; 6 Medical Unit Speech and Language Pathology and Department of Plastic Surgery and Craniofacial Surgery, Karolinska University Hospital, Stockholm, Sweden; 7 Department of Plastic and Maxillofacial Surgery, Uppsala University Hospital, Uppsala, Sweden; 8 Department of Otorhinolaryngology, Division of Speech and Language Pathology, Sahlgrenska University Hospital, Gothenburg, Sweden; 9 Department of Plastic Surgery, Sahlgrenska University Hospital, Gothenburg, Sweden; 10 Department of Speech-Language Pathology, Uppsala University Hospital, Uppsala, Sweden; 11 Department of Plastic Surgery and Craniofacial Surgery, Karolinska University Hospital, Stockholm, Sweden; 12 Speech and Language Therapy Unit, Linköping University Hospital, Linköping, Sweden

**Keywords:** Cleft lip and palate, Registry, Surgery, Speech

## Abstract

**Background:**

The objective of the Swedish cleft lip and palate registry (CLP registry) is to promote quality control, research and improvement of treatment, by the comparison of long-term results. The aim was to compare data from the CLP registry among the six treatment centres, regarding data on surgery and speech outcomes at 5 years of age.

**Methods:**

The participants were 430 children born in Sweden from 2009 to 2014, with cleft palate with or without cleft lip and without known syndromes and/or additional malformations. The number of primary and secondary palatal surgeries up to 5 years of age, timing of the last primary palatal surgery, percentage consonants correct, percentage non-oral speech errors and perceived velopharyngeal competence at 5 years were assessed. Multivariable binary logistic regression adjusted for sex and cleft type was used to compare results between the six centres.

**Results:**

At one centre (centre 4), the palate was closed in one to three stages, and at the remaining centres in one or two stages. At centre 4, more children underwent a higher number of palatal surgeries, and the last primary palatal surgery was performed at a higher age. Children in centre 4 were also less likely to achieve ≥86% correct consonants (OR = 0.169, *P* = < 0.001), have no non-oral speech errors (OR = 0.347, *P* = < 0.001), or have competent or marginally incompetent velopharyngeal competence (OR = 0.244, *P* = < 0.001), compared to the average results of the other centres. No clear association between patient volume and speech outcome was observed.

**Conclusions:**

The results indicated the risk of a negative speech result if the last primary palatal surgery was performed after 25 months of age. Whether the cleft in the palate was closed in one or two stages did not affect speech outcome. The Swedish CLP registry can be used for open comparisons of treatment results to provide the basis for improvements of treatment methods. If deviating negative results are seen consistently at one centre, this information should be acted upon by further investigation and analysis, making changes to the treatment protocol as needed.

**Supplementary Information:**

The online version contains supplementary material available at 10.1186/s12887-022-03367-2.

## Background

In Sweden, about one in 500 children are born with cleft lip with or without cleft palate (CL/P), which on average gives 175 births annually. These children receive treatment by a multidisciplinary team at one of six regional cleft lip and palate (CLP) centres, all connected to the Swedish quality registry for patients born with CL/P (CLP registry). The CLP registry was initiated to enable the continuous evaluation of treatment results at the Swedish CLP centres, with the objective to promote quality control, research and improvements in treatment, via the comparison of long-term results [1].

There are various procedures for primary cleft palate surgery [2]. The procedures differ regarding timing, staging and technique. Speech development benefits from early closure of the hard palate [3, 4], whereas maxillary growth may benefit from delayed closure of the palate [4, 5]. Today, two-stage palatal closure with delayed hard palate closure is used at four of six Swedish CLP centres, with the objective of promoting maxillary growth [5]. At the other two Swedish CLP centres, the palate is closed in one stage.

Surgical protocols for primary palatal surgery have a high degree of diversity and poor evidence base [6]. An aggravating circumstance for research in the area is that the population with cleft palate with or without cleft lip (CP ± L) is small and heterogeneous. Therefore, it may take a long time to collect data of larger groups of children. Timing, techniques for surgery and methods and materials for data collection may then change over time and violate the standardised evaluation of treatment outcome [7]. Multi-centre studies, such as the Scandcleft randomised trials [6] and the TOPS trial [8], allow for the recruitment of larger data sets during a period of time where these variables are kept constant.

However, in randomised controlled trials requiring the participating surgeons to master a new surgical technique, a learning curve could be expected, which may influence the results [9]. This raises ethical issues. Other challenges may include the recruitment of patients at CLP centres where the annual case load is low, and rules of research governance, which may increase the associated costs [9].

Four hundred and forty-eight children born with non-syndromic unilateral cleft lip and palate (UCLP) participated in the Scandcleft trials. In all trials, lip and soft palate closure at 3–4 months and hard palate closure at 12 months was the common method. In trial 1, this method was compared with lip and soft palate closure at 3–4 months and hard palate closure at 36 months, in trial 2 with lip closure at 3–4 months and hard and soft palate closure at 12 months and in trial 3 with lip and hard palate closure at 3–4 months and soft palate closure at 12 months. Speech and dentofacial development served as the primary outcomes. The only statistically verified finding in the Scandcleft trials was that delaying hard palate closure to 36 months of age is associated with poorer consonant proficiency at 5 years of age [10]. Shaw and Semb [9] concluded “that familiarity and operator skill outweigh the importance of protocol”. Thus, when comparing the treatment results of different protocols for primary palatal surgery, it is better if the surgeons adhere to surgical methods that they are already trained to use.

Nationwide studies/audits allow the inter-centre comparison of outcomes and can contribute to valuable scientific knowledge in the CLP area. This has been done for example in the United Kingdom [11] and New Zealand [12]. The Americleft project [13] covers several centres in the United States. Based on the Americleft project, one study on speech outcome after one versus two stage palatal surgery in children with cleft lip and complete cleft palate has been published [14]. National quality registries for patients born with CL/P facilitate continuous follow-up and evaluation of the results in CLP care. The CRANE database [15] in the United Kingdom (England, Wales, Northern Ireland) has published several studies, of which one focused on maxillary growth and speech in 5-year-olds with UCLP [16]. Although the speech assessment procedures in the Swedish CLP registry [1] are based on the same principles for speech analysis [17, 18] as those in the CRANE database [19], the speech outcome measures differ and are not comparable. The Norwegian CLP registry uses the same speech outcome measures [20] as the Swedish CLP registry [1] to a high degree. In the future, it may be possible to compare speech data between the Swedish and Norwegian CLP registries.

Several studies have assessed the reliability of speech data in the Swedish CLP registry [21–23]. Brunnegård et al. [22] assessed the reliability of data on perceived velopharyngeal competence in the CLP registry, which was good to excellent. Furthermore, data on percentage consonants correct and percentage non-oral speech errors have been proven to be satisfying [22, 23]. Data from the Swedish CLP registry now allow for open comparison between CLP centres. The aim of the current study was to compare surgical treatment up to 5 years of age and speech outcomes at 5 years of age using data from the Swedish CLP registry.

## Methods

### Design and setting

All participants were treated and data registered at one of six regional CLP centres, located at university hospitals in Sweden. For analysis, data were retrieved from the CLP registry via Record Centre South, Lund, Sweden. All Swedish CLP centres are connected to the CLP registry and the coverage degree is above 90% [1].

### Participants

**Fig. 1:**
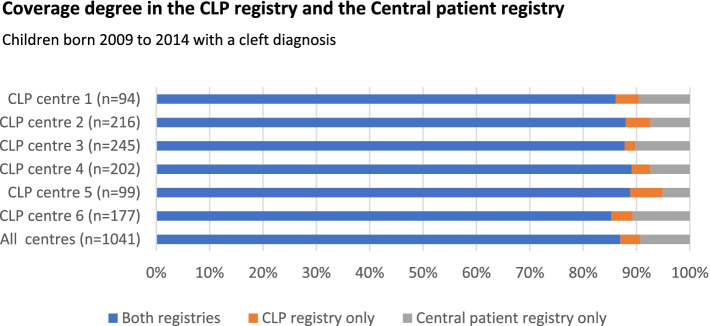
Coverage degree in the Swedish cleft lip and palate (CLP) registry

Children born from 2009 to 2014 with CP ± L (with ICD-10 codes Q35.3 Cleft soft palate, Q35.5 Cleft hard palate with cleft soft palate, Q37.4 Cleft hard and soft palate with bilateral cleft lip, Q37.5 Cleft hard and soft palate with unilateral cleft lip [24]) registered in the CLP registry participated in this study. Children who had been transferred between treatment centres before the 5-year speech registration, children born abroad and children with known additional malformations, syndromes and/or developmental disorders were excluded. For the total number of children born from 2009 to 2014 with CL/P, see Fig. 1.

### Registration of baseline data

At the first visit/patient contact the following data are registered: born in Sweden or not, cleft type (ICD-10 diagnosis [24]) and additional deformities and/or syndromes. For further information, see Klintö et al. [1]. A manual review of the baseline data took place every year and before analysis, to ensure that data were correct.

### Surgical protocols

In Table 1 surgical protocols for primary palatal closure, average annual case load and number of chief operators at different CLP centres are presented. Children with cleft lip or cleft lip and alveolus also underwent primary lip-plasty with simultaneous correction of the nasal cartilage at 3 to 6 months of age. If there was a cleft alveolus, the residual cleft in the alveolar ridge was or should be closed in the mixed dentition at 7 to 11 years of age, by a cancellous bone graft harvested either from the iliac crest or the tibia.

**Table 1:** Surgical protocols for primary palatal closure, average annual case load and number (No.) of chief operators at different cleft lip and palate (CLP) centres

CLP centre	Surgical protocol for primary palatal closure	Average annual CLP case load 2009 to 2014	No. of chief operators
1	One-stage closure with muscle reconstruction according to Sommerlad [25] for SP and SHP. In other cases, two stages modified hybrid technique with muscle dissection according to Sommerlad [25] and minimal lateral incisions.	15.7	1
2	Usually two-stage closure with muscle reconstruction according to Sommerlad [25].	36	3
3	During the period, there was a transition from one-stage closure, mostly using a minimal incision technique with muscle reconstruction [26], to two-stage closure according to a new modified version of the Gothenburg method [27].	40.8	4
4	Usually two-stage closure with a new modified version of the Gothenburg method [27].	33.7	3
5	One-stage closure according to Bardach [28].	16.5	2
6	One-stage closure with muscle reconstruction according to Sommerlad [25].	29.5	1

*SP* cleft soft palate, *SHP* cleft soft and hard palate

### Registration of surgical data

Surgical data were recorded continuously. All operations were coded according to the Swedish National Board of Health and Welfare’s classification of health intervention [29]. Surgical procedures registered were hard and soft palate closure, soft palate closure, hard palate closure, repair of fistula, re-repair of palate, pharyngeal flap, plastic operation of pharynx and buccal flap. In addition, the procedures were recorded as primary or secondary surgery and by which anatomical structure the surgery was related to. If combined surgery, the procedure used was recorded with a major or minor surgical code. Secondary palatal surgery was defined as repair of fistula and re-repair of the palate. In addition, soft palate closure, hard palate closure, hard and soft palate closure, coded as secondary palatal repair when performed a second time, were attributed to this category. Furthermore, speech improving surgery, such as, pharyngeal flap, buccal flap and plastic operation of the pharynx, were included in the term secondary palatal surgery. For further information, see Klintö et al. [1]. A manual review of the surgical data took place every year and before analysis, to ensure that data were correct.

### Assessment of coverage degree and reporting degree of surgical data

Coverage degree was checked by comparing the number of children born from 2009 to 2014 in the CLP registry, with the number of children born from 2009 to 2014 with a cleft diagnosis according to the ICD-10 [24] in the Central patient registry, managed by the Swedish National Board of Health and Welfare.

Reporting degree of cleft-related surgeries for the children participating in the study was assessed by comparing the number of cleft-related surgical intervention codes in the CLP registry for each individual, with the number of surgical intervention codes in the Central patient registry, according to a classification system of the Swedish National Board of Health and Welfare [29], for each individual up to 5 years of age.

### Speech assessment and registration of speech data

A speech-language pathologist specialised in cleft palate speech documented the child’s speech with audio recordings at 5 years of age +/− 6 months. The speech-language pathologists then performed perceptual assessment from the standardised audio recordings, according to the assessment procedure of The Swedish Articulation and Nasality Test (SVANTE) [30]. The speech variables registered were perceived velopharyngeal competence, percentage consonants correct and percentage non-oral speech errors. Perceived velopharyngeal competence, i.e., an overall assessment of hypernasality, audible nasal air leakage and weak articulation, was rated on a three-point scale with the scale values ‘competent/sufficient’, ‘marginally incompetent/insufficient’ and ‘incompetent/insufficient’ [30]. The speech-language pathologists also performed phonetic transcriptions according to the International Phonetic Alphabet [31] for the 59 target consonants in SVANTE and calculated the percentage consonants correct and percentage non-oral speech errors [30]. For further information on speech data in the CLP registry, see Klintö et al. [1]. A manual review of the speech data took place every year and before analysis, to ensure that data were correct.

### Statistical analysis

Patient characteristics of participants and excluded children were compared using the Pearson χ^2^-test.

Results of the binary quality indicator ≥86% correct consonants, based on the variable percentage consonants correct [23], were analysed statistically. The children were dichotomised into groups with and without ≥86% correct consonants. The cut-off corresponds to − 2 SD from the mean in norm data of Swedish-speaking 5-year-olds without CLP [30]. Results of the binary quality indicator Without non-oral speech errors, based on the variable percentage non-oral speech errors [22], were also analysed. For this quality indicator, a maximum of 5% non-oral speech errors was allowed, as a margin of errors. Also, the results of the binary quality indicator Competent or marginally incompetent velopharyngeal function [22] were analysed, based on the three-point scale for rating of perceived velopharyngeal competence [30].

Multivariable binary logistic regression was used to compare differences in speech results between the six centres. Outcome was presented by odds ratios (OR) of positive results (i.e., ≥ 86% correct consonants, Without non-oral speech errors, Competent or marginally incompetent velopharyngeal competence) with corresponding 95% confidence intervals. A large centre contributes strongly to the grand mean of all centres, and deviations from the grand mean would hence be difficult to detect for large centres. Therefore, the result of each centre was compared with the overall (mean) results of the other centres (effect coding). Hence, an OR > 1 indicates that children in that centre had a higher chance of a positive speech result than children in the other centres. In addition to centre, the model included the independent variables sex and diagnosis to adjust for differences in the patient case-mix.

For all statistical analyses, *P* < 0.05 (2-tailed) was considered to indicate significant differences. SAS software, Version 9.4 of the SAS System for Windows (SAS Institute Inc., Cary, NC, USA) was used for analyses.

## Results

### Missing data

The inclusion criteria were fulfilled by 472 children. Of these, seven did not come for the 5-year follow-up (three from centre 3, three from centre 4 and one from centre 6). Four were excluded as they did not speak during the assessment (two from centre 3, one from centre 4 and one from centre 5). Furthermore, 31 children were excluded as they had been registered outside the time span of +/− 6 months at 5 years (one from centre 1, 17 from centre 2, one from centre 3, two from centre 4, eight from centre 5 and two from centre 6). This resulted in a total of 430 children. The excluded patients did not differ significantly from the participants regarding primary diagnosis (*P* = 0.742), sex (*P* = 0.399), number of stages for primary palatal surgery (*P* = 0.623), age of the child when the last primary palatal surgery was performed (*P* = 0.141), or if secondary surgery had been performed or not (*P* = 0.766) (Additional file 1). The number of included participants from each centre varied largely, from 36 participants to 101 (Table 2).

**Table 2:** Multivariable logistic regression modelling the odds of “positive results”

	N	≥ 86%% correct consonants		Without non-oral speech errors		Competent or marginally incompetent velopharyngeal function	
		OR	(95% CI of OR)	OR	(95% CI of OR)	OR	(95% CI of OR)
Centre 1	36	1.503	(0.743–3.040)	1.334	(0.472–3.771)	1.332	(0.471–3.765)
Centre 2	84	0.965	(0.606–1.537)	1.136	(0.582–2.216)	1.093	(0.561–2.132)
Centre 3	101	0.957	(0.616–1.486)	0.721	(0.413–1.258)	**2.652**	(1.172–5.995)
Centre 4	78	**0.169**	(0.098–0.292)	**0.347**	(0.196–0.612)	**0.244**	(0.137–0.434)
Centre 5	51	**2.556**	(1.331–4.909)	0.879	(0.413–1.868)	1.020	(0.466–2.233)
Centre 6	80	1.663	(0.994–2.782)	**3.002**	(1.217–7.406)	1.039	(0.541–1.994)
Boys	234	Ref.					
Girls	196	**2.061**	(1.268–3.349)	1.363	(0.726–2.560)	1.380	(0.718–2.651)
UCLP	149	Ref.					
BCLP	77	**0.262**	(0.136–0.507)	**0.305**	(0.146–0.637)	0.462	(0.201–1.064)
SHP	148	1.450	(0.823–2.556)	0.678	(0.311–1.480)	**0.397**	(0.178–0.885)
SP	56	**3.647**	(1.621–8.207)	1.769	(0.556–5.634)	2.267	(0.609–8.437)

Odds ratio (OR) with 95% confidence intervals (CI) compared to the average result of the other centres, or a reference category (Ref.) corresponding to “Boys” and “UCLP”. Statistically different ORs are marked in bold

*UCLP* unilateral cleft lip and palate, *BCLP* bilateral cleft lip and palate, *SHP* cleft soft and hard palate, *SP* cleft soft palate

### Coverage degree and reporting degree of cleft-related surgeries

**Fig. 2:**
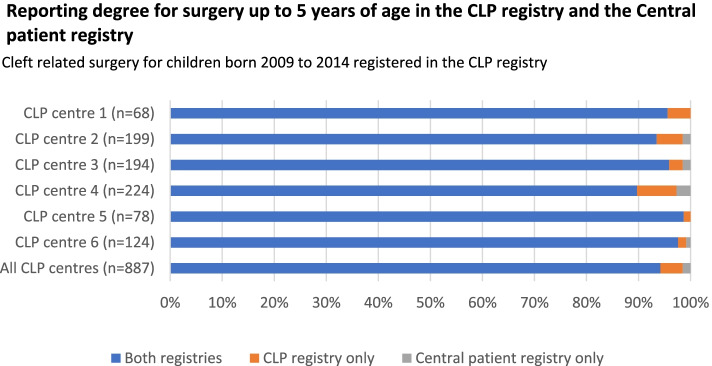
Reporting degree for cleft related surgeries in the Swedish cleft lip and palate (CLP) registry

Average coverage degree in the CLP registry for all children with CLP in Sweden born 2009 to 2014 was 90.7% (Fig. 1). Average reporting degree for cleft-related surgeries in the CLP registry for children participating in the present study was 98.5%, and the reporting degree was above 97% at all centres (Fig. 2).

### Background data of participating children

**Fig. 3:**
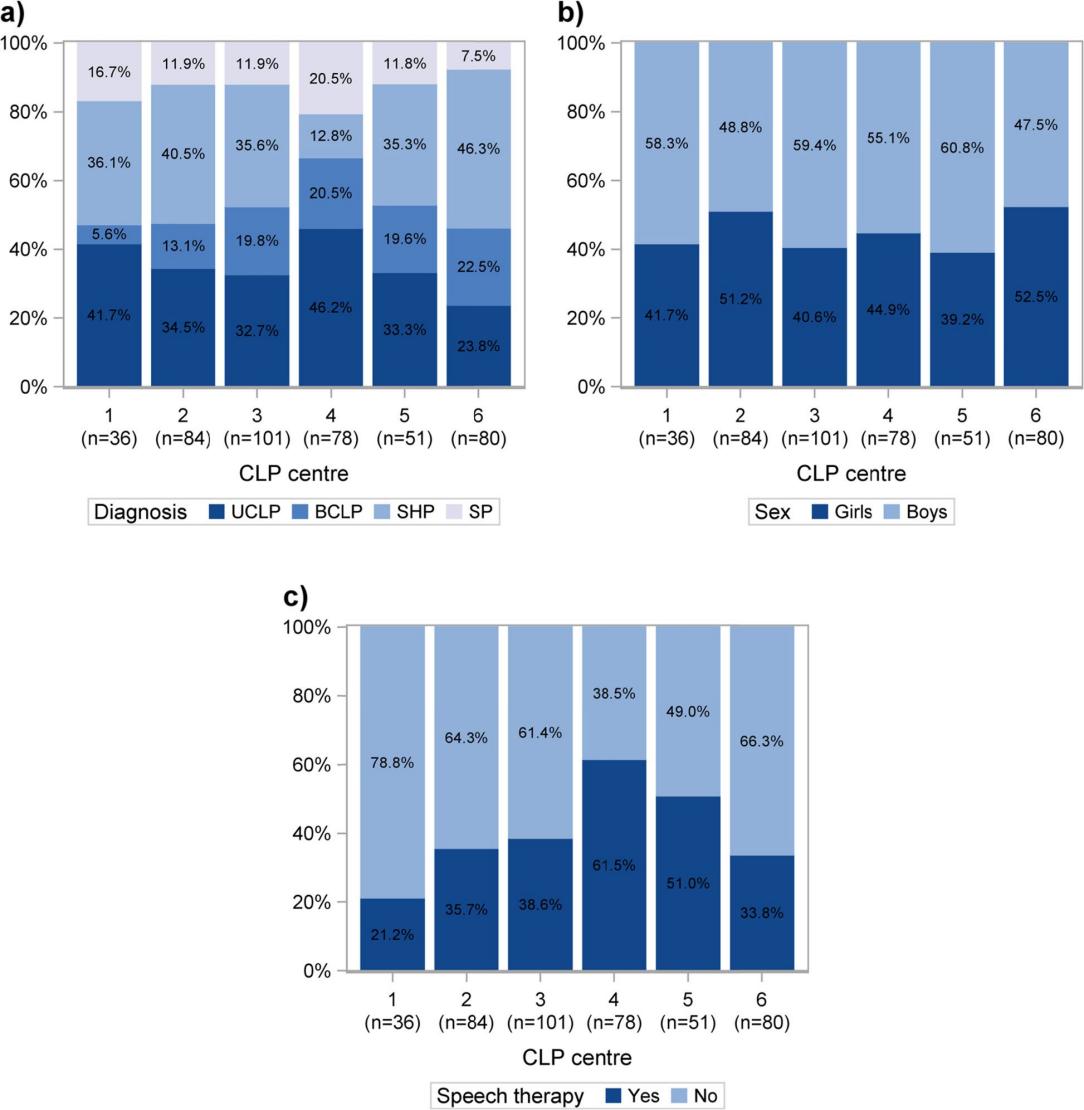
Distribution of primary diagnoses (**a**) and sex (**b**) among participating children, and proportion of children who had undergone speech therapy (**c**) at each cleft lip and palate (CLP) centre. UCLP = unilateral cleft lip and palate, BCLP = bilateral cleft lip and palate, SHP = cleft soft and hard palate, SP = cleft soft palate

The distribution of primary cleft diagnoses according to ICD-10 [24] varied at different CLP centres (Fig. 3a). SP was least common at centre 6 (7.5%) and most common at centre 4 (20.5%). SHP was least common at centre 4 (12.8%) and most common at centre 6 (46.3%). Bilateral cleft lip and palate (BCLP) was least common at centre 1 (5.6%) and most common at centre 6 (22.5%). UCLP was least common at centre 6 (23.8%) and most common at centre 4 (46.2%) (Fig. 3a). The distribution of sex varied slightly at different centres (Fig. 3b). The lowest proportion of girls was seen at centre 5 (39.2%) and the highest at centre 6 (52.5%) (Fig. 3b).

The proportion of children who had undergone speech therapy with a speech-language pathologist before 5 years of age varied largely among centres (Fig. 3c). The lowest proportion of children who had undergone speech therapy was seen at centre 1 (21.2%) and the highest at centre 4 (61.5%) (Fig. 3c).

### Surgery up to 5 years of age

**Fig. 4:**
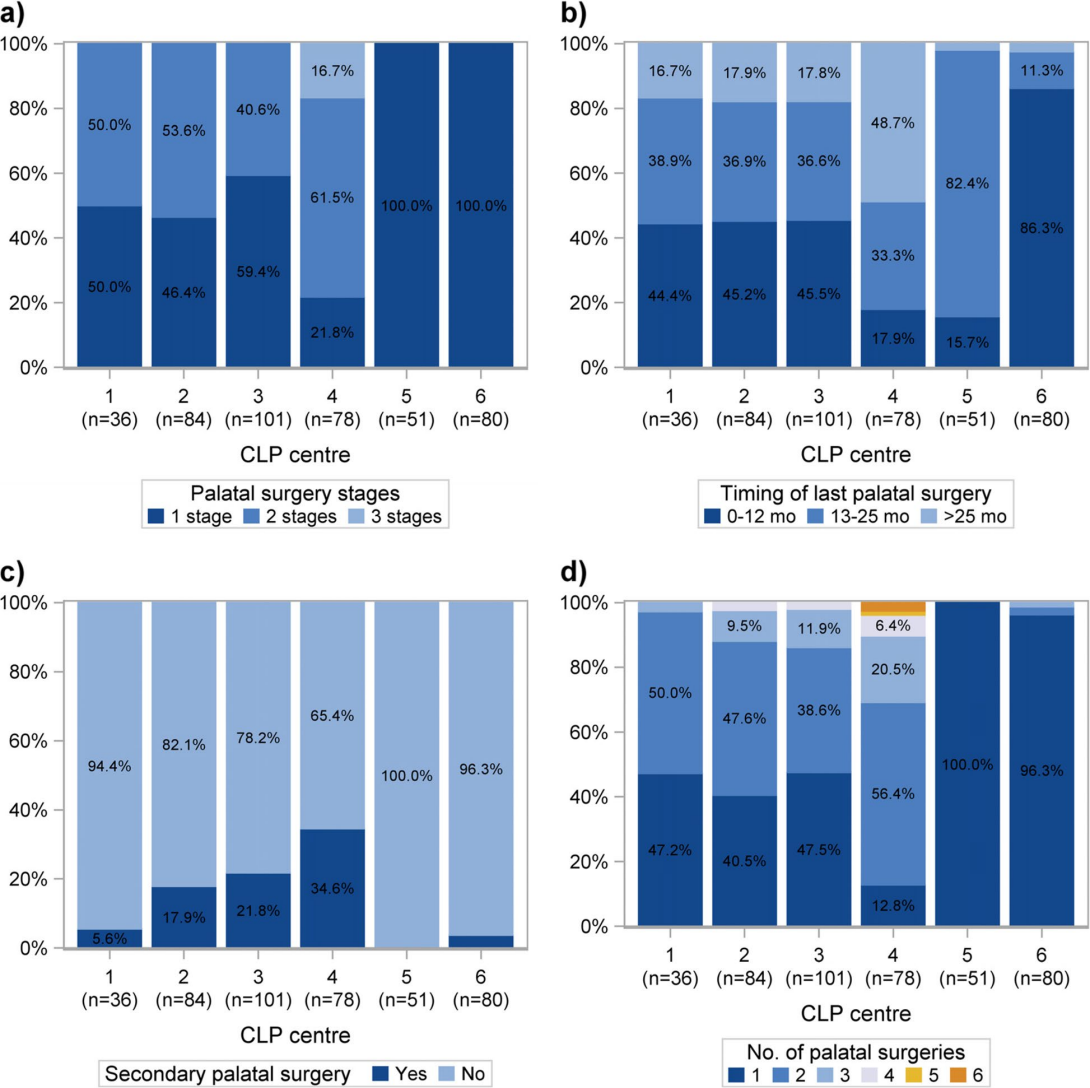
Number of stages for primary palatal surgery (**a**), the child’s age in months (mo) when the last primary palatal surgery was performed (**b**), occurrence of secondary palatal surgery (**c**) and number of occasions with palatal surgery (**d**) at each cleft lip and palate (CLP) centre. The bars correspond to the proportion of children

At centres 1, 2 and 3, the children had been treated with primary palatal surgery in one or two stages (Fig. 4a). At centre 4, primary palatal surgery had been performed in one, two or three stages, with two stage surgery being the most common. At centres 5 and 6, all children had been treated with primary palatal surgery in one stage (Fig. 4a).

At centres 1, 2 and 3, the timing of the last primary palatal surgery for about 45% of the children was before 13 months of age, for 36.6 to 38.9% between 13 and 25 months of age and for 16.7 to 17.9% after 25 months of age (Fig. 4b). At centre 4, the most common timing of the last primary palatal surgery (in 48.7% of the cases) was after 25 months of age. At centre 5, the timing of the last primary palatal surgery for most children (82.4%) was between 13 and 25 months of age and at centre 6 the most common timing (86.3%) was before the age of 13 months (Fig. 4b). At centre 5, no children had been treated with secondary palatal surgery (Fig. 4c). The highest proportion of children (34.6%) treated with secondary palatal surgery was seen at centre 4 (Fig. 4c).

In Fig. 4d, the number of occasions of palatal surgery up to 5 years of age is presented. At centre 5, all children had undergone palatal surgery only once. The greatest number of palatal surgeries were performed at centre 4, where most children underwent palatal surgery two (56.4%), or three (20.5%) times, but eight children underwent surgery four to six times (Fig. 4d).

### Speech outcome at 5 years of age

**Fig. 5:**
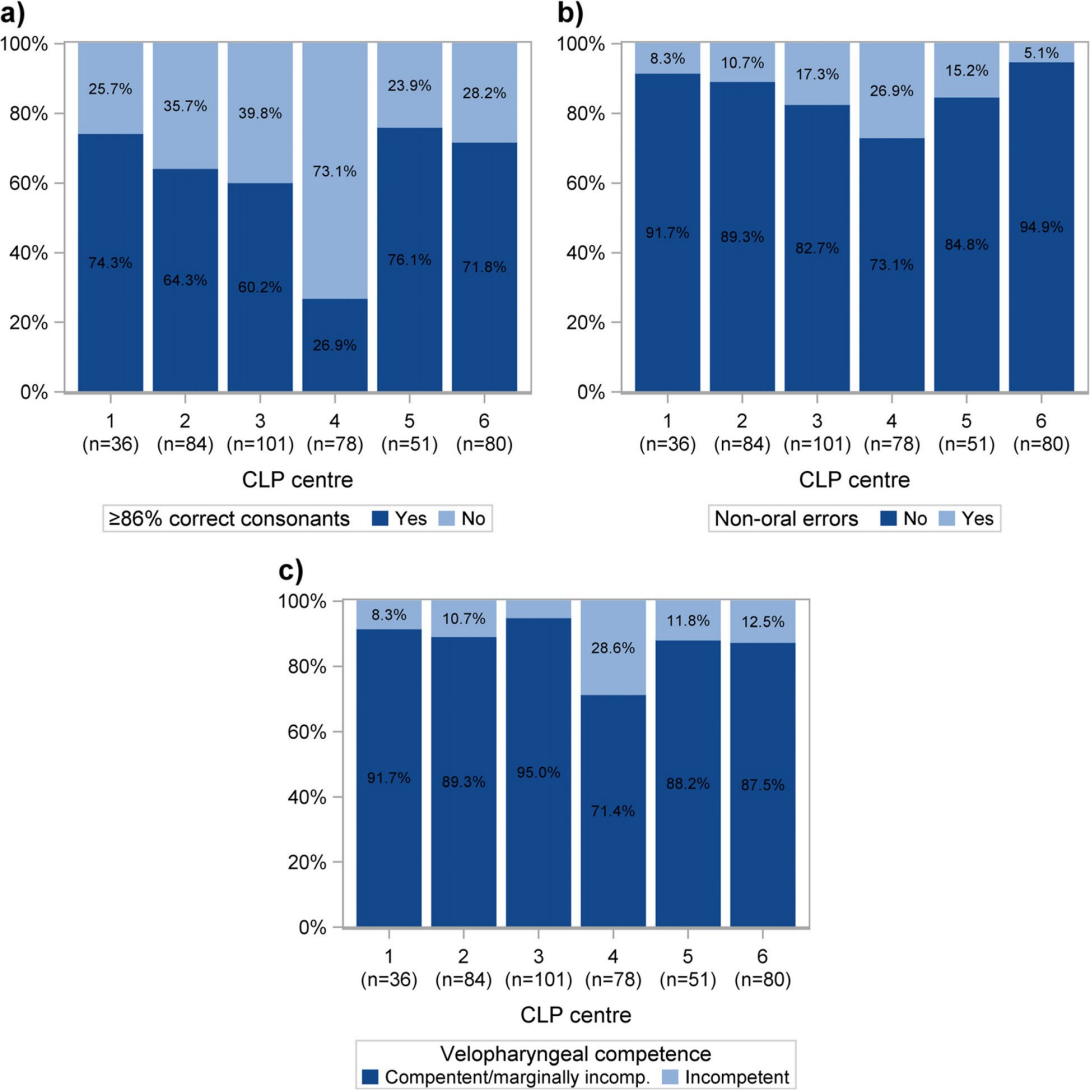
Proportion of children at each cleft lip and palate (CLP) centre with/without > 86% correct consonants (**a**), non-oral speech errors (**b**) and competent/marginally incompetent velopharyngeal competence (**c**)

At 5 out of 6 CLP centres (1, 2, 3, 5, 6), 60% or more of the children had ≥86% correct consonants (Fig. 5a). At centre 4, 26.9% of the children had ≥86% correct consonants (Fig. 5a). At 5 out of 6 CLP centres (1, 2, 3, 5, 6) more than 80% of the children displayed no non-oral speech errors (Fig. 5b). At centre 4, 73.1% of the children displayed no non-oral speech errors (Fig. 5b). At 5 out of 6 CLP centres (1, 2, 3, 5, 6) more than 85% of the children had competent or marginally incompetent velopharyngeal function (Fig. 5c). At centre 4, 71.4% of the children had competent or marginally incompetent velopharyngeal function (Fig. 5c).

Speech results differed between girls and boys and between diagnoses (Table 2). Girls were more likely than boys to achieve ≥86% correct consonants (OR = 2.061, *P* = 0.003), while the difference in non-oral speech errors (*P* = 0.335) and velopharyngeal competence (*P* = 0.334) did not differ significantly. Children with BCLP were less likely to achieve ≥86% correct consonants (OR = 0.262, *P* = < 0.001) and have no non-oral speech errors (OR = 0.305, *P* = 0.002) than children with UCLP. Children with SP were more likely to achieve ≥86% correct consonants (OR = 3.647, *P* = 0.002) compared to children with UCLP. Children with SHP were less likely to have competent or marginally incompetent velopharyngeal competence compared to children with UCLP (OR = 0.397, *P* = 0.0239).

Centre comparisons were performed for the quality indicators Competent or marginally incompetent velopharyngeal competence, ≥ 86% correct consonants and Without non-oral speech errors, adjusted for centre differences in the distribution of boys and girls and cleft type (Table 2). Children in centre 4 were less likely to achieve ≥86% correct consonants (OR = 0.169, *P* = < 0.001), have no non-oral speech errors (OR = 0.347, *P* = < 0.001), or have competent or marginally incompetent velopharyngeal competence (OR = 0.244, *P* = < 0.001) compared to the average results of the other centres. The children were more likely to achieve ≥86% correct consonants at centre 5 (OR = 2.556, *P* = 0.005), to have no non-oral speech errors at centre 6 (OR = 3.002, *P* = 0.017), and to have competent or marginally incompetent velopharyngeal competence at centre 3 (OR = 2.652, *P* = 0.019), compared to the average results the other centres. We observed no clear association between patient volume and speech outcome (Tables 1 and 2).

## Discussion

Surgical procedures up to 5 years of age and speech outcomes at 5 years of age varied between the six Swedish CLP centres. At centre 4, where the palate was closed in more stages in several cases than at the other centres, the children underwent a higher number of cleft palate-related surgeries, with complete primary palate closure at a higher age; they also showed less favourable speech outcome on average. At centre 5 children were more likely to achieve ≥86% correct consonants, at centre 6 to have no non-oral speech errors, and at centre 3 to have competent or marginally incompetent velopharyngeal competence compared to the average results of the other centres.

The results indicate that it does not matter whether the last primary palatal surgery is performed before 13 months or between 13 and 25 months of age for the speech results. At centre 6 the palate in most cases was closed in one stage before 13 months of age, at centres 1, 2, and 3 most children had their last primary palatal surgery before 13 months or between 13 and 25 months of age, and at centre 5 a major part of the children were treated with one stage closure of the palate between 13 and 25 months of age. At these five centres, no deviating negative speech results were seen. However, at centre 4, where a major part of the children had their last primary palatal surgery after 25 months of age, the children were more likely to have a negative speech result at 5 years of age. This is in line with findings of the Scandcleft trials, where delaying hard palate closure to 36 months of age was associated with poorer consonant proficiency at 5 years of age [10].

According to the surgical protocols at all six Swedish CLP centres, the last primary palatal surgery should be performed at 24 months of age at the latest. Sometimes, surgery is delayed due to the child’s health condition, or organisational reasons, such as a lack of operating rooms, anaesthesiologists and surgical nurses. In centre 4, a delay was also related to more complications that required additional secondary surgical treatment associated with the new method of soft palate closure that was introduced in 2008. However, this method was also abandoned in 2014. Nevertheless, the results of the present and previous studies [10] are important when decisions are made to delay the primary palate surgery in children with cleft palate and when prioritising among patients awaiting surgery. Delaying palatal closure may require more healthcare resources in the long-term. In the present study, the centre with the highest proportion of children who underwent the last primary palatal surgery after 25 months of age, had a higher proportion of children who underwent secondary palatal surgery before the age of 5. As previously mentioned, this was due to a new modified method of palate repair with a high frequency of complications leading to poor velopharyngeal function. In addition, this centre also had a higher proportion of children who underwent speech-language therapy before 5 years of age.

As in previously published studies comparing speech outcome after one and two stage primary palatal surgery [10, 14, 32], the results of the present study indicated that whether the cleft in the palate is closed in one or two stages does not affect speech outcome. At two centres (5, 6) out of the five centres with no deviating negative speech results at 5 years of age, the palate was closed in one stage. At the other three (1, 2, 3) the palate was closed in two stages in 40.6 to 53.6% of the cases, and in one stage in the remaining cases. At centre 4, where the children were more likely to have negative speech results at 5 years of age, primary palatal surgery was performed in two stages in 61.5% of the cases and in three stages in 16.7% of the cases. Furthermore, as described above, at centre 4 the last primary palatal surgery was performed at a later age than at the other centres. It cannot be ruled out that both the later timing of the last primary palatal surgery and complications regarding the surgical technique, resulting in up to three stages of primary palatal closure, had a negative effect on speech.

The objective of two-stage palatal surgery is to promote maxillary growth [5]. In the Scandcleft trials, no differences in the dental arch relationships of children were seen at 5, 8 and 10 years of age related to different surgical protocols [33]. The final outcomes of maxillary growth can only be evaluated when growth is completed, in adulthood. This will be investigated in future studies based on the Swedish CLP registry.

In the present study, children with BCLP were less likely to achieve ≥86% correct consonants and have no non-oral speech errors and children with SP more likely to achieve ≥86% correct consonants, compared to children with UCLP. This finding is in accordance with results in previous studies, where more extensive orofacial clefts have been associated with poorer speech [34, 35]. Children with SHP were less likely to have competent or marginally incompetent velopharyngeal function than children with UCLP. A tendency of poorer velopharyngeal function in 5-year-olds with SHP compared to peers with other cleft types was seen in an earlier study, although the subgroups were small, and no significant differences were seen between groups [35]. Furthermore, girls were more likely than boys to achieve ≥86% correct consonants. This could be explained by the fact that cleft palate involving the lip is more common in males and cleft palate only in females [36], with the risk of consonant errors being higher in cases with more extended clefts.

A quality registry with high coverage and reporting degree may be an effective means for continuous open comparisons and knowledge-based management of CLP care. At centre 4, a new modified surgical method was used for closure of the palate of children born from 2009 to 2014. By evaluating the results of surgery and speech and comparing the results with those of the other Swedish CLP centres, it was clarified that the modified surgical method resulted in poorer postoperative speech results. Due to the poor results with high complication rate resulting in later closure of the palate when using the modified Gothenburg method, the method was changed for children born in 2015 and after. The original method of primary palatal surgery has therefore been/is used in both centre 4 and centre 3 since then. Open comparisons of data from the Swedish CLP registry are carried out every year and the effect of reverting to the original method at centres 4 and 3 will be evaluated. This is an example of how quality registries for CLP care may be used.

Forty-two children were excluded from the study. They did not differ significantly from participants, and no resulting bias is likely to have affected our findings. Several factors may contribute to the variations in speech results at different CLP centres, such as differences in treatment methods, coding of diagnoses and surgery and speech assessment. Within the Swedish CLP registry network, continuous data validation and calibration of registry users are ongoing [1]. However, it cannot be excluded that differences in assessment and coding may have affected the results of this study, since it was based on retrospective data. Although the reliability of the registered speech data has generally been proven to be good [22, 23], there may be differences in how strict different speech-language pathologists are when assessing different speech variables. Therefore, if deviant results are discovered when evaluating retrospective data, it is important to go back and scrutinise the raw data. If the deviating results remain, one needs to try to remedy the causes of the deviating results, as done at centre 4.

The centralisation of cleft care has been proposed as an intervention for the improvement of cleft care [11]. In the United Kingdom, the number of CLP centres was reduced from 57 to 11, to improve the treatment outcomes. Surgeons at these 11 CLP centres operate on at least 35 cases annually. The outcomes have improved post-centralisation [11]. In the present study, no clear association between patient volume and speech outcome was observed. Centres with fewer patients than 30 per year (1, 5, 6) and centres with more than 35 patients per year (2, 3) showed broadly equivalent results. One explanation for this may be that it is not only the caseload of a CLP centre that affects the outcomes, but also other factors, such as how many surgeons are active at a centre and routines for training inexperienced surgeons. The number of chief operators varied between one and four at different centres in the present study. In addition, the availability of anaesthesiologists, surgical nurses and surgery theatres are important, as it affects whether the established surgery protocol can be followed. The results highlight the importance of a well-functioning organisation of CLP care.

## Conclusions

The results indicated a risk of a negative speech result if the last primary palatal surgery was performed after 25 months of age. Whether the cleft in the palate was closed in one or two stages did not affect speech outcome. The Swedish CLP registry is useful for the open comparison of treatment results, to provide a basis for the improvement of treatment methods. If deviating negative results are seen consistently at a centre, one should act on this information by further investigation and analysis and making changes to the treatment protocol as needed.

## Supplementary Information

**Additional file 1: Table 1**. Comparisons of group characteristics for participants and excluded children. Proportions, and *P*-values from a χ^2^_-_test.

## Data Availability

The datasets generated and/or analysed during the current study are not publicly available due ethical restrictions but are available from the corresponding author on reasonable request. Additionally, To access the data in the Swedish CLP registry, approval from an Ethics Board and Region Skåne is required (https://vardgivare.skane.se/kompetensutveckling/forskning-inom-region-skane/utlamnande-av-patientdatasamradkvb/). Lists of included variables and results published in annual reports may be retrieved in Swedish from the website of the CLP registry: http://lkg-registret.se/. An overview of the included data can be provided by The Swedish Research Council: https://www.registerforskning.se/en/registers-in-sweden/easier-to-find-register-data-with-the-register-utiliser-tool/.

## Abbreviations

BCLP: Bilateral cleft lip and palate
CI: Confidence interval
CLP: Cleft lip and palate
CL/P: Cleft lip with or without cleft palate
CP ± L: Cleft palate with or without cleft lip
OR: Odds ratio
SHP: Cleft soft and hard palate
SP: Cleft soft palate
SVANTE: The Swedish articulation and nasality test
UCLP: Unilateral cleft lip and palate
VPC: Perceived velopharyngeal competence

## References

[1] Klintö K, Karsten A, Marcusson A, Paganini A, Rizell S, Cajander J (2020). Coverage, reporting degree and design of the Swedish quality registry for patients born with cleft lip and/or palate. BMC Health Serv Res.

[2] Leow AM, Lo LJ (2008). Palatoplasty: evolution and controversies. Chang Gung Med J.

[3] Peterson-Falzone SJ (1996). The relationship between timing of cleft palate surgery and speech outcome: what have we learned, and where do we stand in the 1990s?. Semin Orthod.

[4] Rohrich RJ, Love EJ, Byrd HS, Johns DF (2000). Optimal timing of cleft palate closure. Plast Reconstr Surg.

[5] Friede H (2007). Maxillary growth controversies after two-stage palatal repair with delayed hard palate closure in unilateral cleft lip and palate patients: perspectives from literature and personal experience. Cleft Palate Craniofac J.

[6] Semb G, Enemark H, Friede H, Paulin G, Lilja J, Rautio J (2017). A Scandcleft randomised trials of primary surgery for unilateral cleft lip and palate: 1. Planning and management. J Plast Surg Hand Surg.

[7] Lohmander A, Howard S, Lohmander A (2011). Surgical intervention and speech outcomes in cleft lip and palate. Cleft palate speech: assessment and intervention.

[8] Conroy EJ, Cooper R, Shaw W, Persson C, Willadsen E, Munro KJ (2021). A randomised controlled trial comparing palate surgery at 6 months versus 12 months of age (the TOPS trial): a statistical analysis plan. Trials..

[9] Shaw W, Semb G (2017). The Scandcleft randomised trials of primary surgery for unilateral cleft lip and palate: 11. What next?. J Plast Surg Hand Surg.

[10] Willadsen E, Lohmander A, Persson C, Lundeborg I, Alaluusua S, Aukner R (2017). Scandcleft randomised trials of primary surgery for unilateral cleft lip and palate: 5. Speech outcomes in 5-year-olds - consonant proficiency and errors. J Plast Surg Hand Surg.

[11] Ness AR, Wills AR, Waylen A, Smallridge J, Hall AJ, Sell D (2018). Closing the loop on centralization of cleft care in the United Kingdom. Cleft Palate Craniofac J.

[12] Morrison MM, Mason NT, Forde BL, Stone PR, Fowler PV, Thompson JMD. Speech outcomes of a national cohort of children with orofacial cleft at 5 and 10 years of age. Cleft Palate Craniofac J. 2021; Online ahead of print. 10.1177/10556656211044939 34672811

[13] Americleft. Americleft. 2018. https://www.americleft.org/aboutus/. Accessed 2 Feb 2022.

[14] Crowley JS, Friesen TL, Gabriel RA, Hsieh S, Wacenske A, Deal D (2021). Speech and audiology outcomes after single-stage versus early 2-stage cleft palate repair. Ann Plast Surg.

[15] The Cleft Registry and Audit NEtwork (2021). Annual report 2021.

[16] Fell M, Medina J, Fitzsimons K, Seifert M, Roberts A, Russell C, et al. The relationship between maxillary growth and speech in children with a unilateral cleft lip and palate at 5 years of age. Cleft Palate Craniofac J. 2021;2021 Online ahead of print. 10.1177/10556656211010620 33887986

[17] Harding A, Grunwell P (1998). Active versus passive cleft-type speech characteristics. Int J Lang Commun Disord.

[18] Hutters B, Brøndsted K (1987). Strategies in cleft palate speech--with special reference to Danish. Cleft Palate J.

[19] Sell D, John A, Harding-Bell A, Sweeney T, Hegarty F, Freeman J (2009). Cleft audit protocol for speech (CAPS-A): a comprehensive training package for speech analysis. Int J Lang Commun Disord.

[20] Norsk kvalitetsregister for leppe-kjeve-ganespalte (2021). Årsrapport 2020 [annual report 2020; Norwegian].

[21] Malmborn JO, Becker M, Klintö K (2018). Problems with reliability of speech variables for use in quality registries for cleft lip and palate-experiences from the Swedish cleft lip and palate registry. Cleft Palate Craniofac J.

[22] Brunnegard K, Hagberg E, Havstam C, Okhiria Å, Klintö K (2020). Reliability of speech variables and speech-related quality indicators in the Swedish cleft lip and palate registry. Cleft Palate Craniofac J.

[23] Klintö K, Hagberg E, Havstam C, Nelli C, Okhiria Å, Brunnegård K. Reliability of data on percent correct consonants and its associated quality indicator in the Swedish cleft lip and palate registry. Accepted for publication in Logopedics Phoniatrics Vocology. 2022. 10.1080/14015439.2022.2095017 35786207

[24] World Health Organization (2016). International statistical classification of diseases and related health problems 10th revision (ICD-10)-WHO version for 2016.

[25] Sommerlad BC (2003). A technique for cleft palate repair. Plast Reconstr Surg.

[26] Mendoza M, Molina F, Azzolini C, Rivera AY (1994). Minimal incision palatopharyngoplasty. A preliminary report. Scand J Plast Reconstr Surg Hand Surg.

[27] Friede H, Lilja J, Lohmander A, Berkowitz S (2013). Two-stage palatal surgery with early veloplasty and delayed hard palate repair: a balanced view on speech and midfacial growth outcome. Cleft lip and palate: diagnosis and management.

[28] Bardach J, Salyer KE, Bardach J (1999). Two-flap palatoplasty: Bardach's technique. Salyer & Bardach's atlas of craniofacial & cleft surgery. Volume II: cleft surgery.

[29] Socialstyrelsen (2004). Klassifikation av kirurgiska åtgärder 1997 (KKÅ) -reviderad 2004 [classification of surgery 1997 (KKÅ) - revised 2004; Swedish].

[30] Lohmander A, Lundeborg I, Persson C (2017). SVANTE - the Swedish articulation and nasality test - normative data and a minimum standard set for cross-linguistic comparison. Clin Linguist Phon.

[31] The International Phonetic Association. The International Phonetic Alphabet and the IPA chart. The International Phonetic Alphabet (2015 revised edition). extIPA Symbols for Disordered Speech (Revised to 2008). 2018/2005. https://www.internationalphoneticassociation.org/content/ipa-chart. Accessed 2 Feb 2022.

[32] Lohmander A, Persson C, Willadsen E, Lundeborg I, Alaluusua S, Aukner R (2017). Scandcleft randomised trials of primary surgery for unilateral cleft lip and palate: 4. Speech outcomes in 5-year-olds - velopharyngeal competency and hypernasality. J Plast Surg Hand Surg.

[33] Heliövaara A, Küseler A, Skaare P, Bellardie H, Mølsted K, Karsten A, et al. Scandcleft randomized trials of primary surgery for unilateral cleft lip and palate: comparison of dental arch relationships and dental indices at 5, 8, and 10 years. Eur J Orthod. 2021;cjab055. 10.1093/ejo/cjab055. 10.1093/ejo/cjab055 PMC9127717 34476476

[34] Choa RM, Slator R, Jeremy A, Robinson S, Franklin D, Roberts A (2014). Identifying the effect of cleft type, deprivation and ethnicity on speech and dental outcomes in UK cleft patients: a multi-centred study. J Plast Reconstr Aesthet Surg.

[35] Klintö K, Falk E, Wilhelmsson S, Schönmeyr B, Becker M (2018). Speech in 5-year-olds with cleft palate with or without cleft lip treated with primary palatal surgery with muscle reconstruction according to Sommerlad. Cleft Palate Craniofac J.

[36] Vu GH, Warden C, Zimmerman CE, Kalmar CL, Humphries LS, McDonald-McGinn D (2022). Poverty and risk of cleft lip and palate: an analysis of United States birth data. Plast Reconstr Surg.

