# Supplementary material for: Inter-centre comparison of data on surgery and speech outcomes at 5 years of age based on the Swedish quality registry for patients born with cleft palate with or without cleft lip
Source: BMC Pediatr. 2022 May 23;22:303. doi: 10.1186/s12887-022-03367-2 (PMC9125901; doi:10.1186/s12887-022-03367-2)
Supplement: Supplementary file 1 — Additional file 1: Table 1. Comparisons of group characteristics for participants and excluded children. Proportions, and P-values from a χ2-test. [file 12887_2022_3367_MOESM1_ESM.docx]

Additional table 1. Comparisons of group characteristics for participants and excluded children. Proportions, and P-values from a χ^2^_-_test.

|  | Participants  (n=430) | Excluded  (n=42) | P-value |
| --- | --- | --- | --- |
| Sex |  |  | 0.399 |
| - Boys (%) | 54.4 | 47.6 |  |
| - Girls (%) | 45.6 | 52.4 |  |
| Diagnosis |  |  | 0.742 |
| - SP (%) | 13.0 | 11.9 |  |
| - SHP (%) | 34.4 | 40.5 |  |
| - BCLP (%) | 17.9 | 11.9 |  |
| - UCLP (%) | 34.6 | 35.7 |  |
| Timing of last palatal surgery |  |  | 0.141 |
| - 0-12 months (%) | 44.4 | 28.6 |  |
| - 13-25 months (%) | 37.0 | 47.6 |  |
| - >25 months (%) | 18.6 | 23.8 |  |
| Palatal surgery stages |  |  | 0.623 |
| - 1 (%) | 61.6 | 54.8 |  |
| - 2 (%) | 35.4 | 42.9 |  |
| - 3 (%) | 3.0 | 2.4 |  |
| Secondary palatal surgery (%) | 16.0 | 14.3 | 0.766 |

SP=cleft soft palate, SHP=cleft soft and hard palate, BCLP=bilateral cleft lip and palate, UCLP=unilateral cleft lip and palate.
